# Characterization of a novel pathogenic variant in the *FECH* gene associated with erythropoietic protoporphyria

**DOI:** 10.1016/j.ymgmr.2019.100481

**Published:** 2019-06-25

**Authors:** Michele C. Kieke, Jacob Klemm, Arthur Rech Tondin, Victor Alencar, Nathan Johnson, Ashley M. Driver, Thomas Lentz, Gregory J. Fischer, Diane A. Caporale, Luke J. Drury

**Affiliations:** a Regions Hospital, Medical Laboratory and Pathology Services, Saint Paul, MN, USA; b University of Wisconsin Stevens Point, Department of Biology, Stevens Point, WI, USA; c PreventionGenetics, Marshfield, WI, USA

**Keywords:** Erythropoietic protoporphyria, Hepatic porphyria, Genetic disease

## Abstract

Erythropoietic protoporphyria (EPP) is an autosomal recessive deficiency in heme biosynthesis due to pathogenic variants in the ferrochelatase gene (*FECH*). Patients present with lifelong photosensitivity and potential liver disease. Here we report a novel *FECH* variant designated c.904_912+1del found in *trans* with the c.315-48T>C hypomorphic variant, in one family with three affected individuals. These patients presented with immediate painful cutaneous photosensitivity but no hepatic manifestations. All have elevated protoporphyrin levels consistent with a diagnosis of EPP. Genetic, biochemical, and functional assay results obtained for this family suggest that the unique variant c.904_912+1del is likely pathogenic and thus causative of EPP.

## 1 Introduction

There are currently six different forms of cutaneous porphyria caused by specific pathogenic variants in genes involved in heme biosynthesis: hereditary coproporphyria (*CPOX*), variegate porphyria (*PPOX*), porphyria cutanea tarda/hepatoerythropoietic porphyria (*UROD*), congenital erythropoietic protoporphyria (*UROS*), erythropoietic protoporphyria (*FECH*), and X-linked protoporphyria/XLP (*ALAS2*). Erythropoietic protoporphyria (EPP) is a rare, inherited autosomal recessive disorder caused by pathogenic variants in the *FECH* gene, which encodes the last enzyme in the heme biosynthesis pathway (ferrochelatase). Interestingly, over 90% of individuals with EPP harbor the same hypomorphic variant (c.315-48T>C) found in *trans* to a second pathogenic variant [1].

The primary clinical manifestation of EPP is cutaneous photosensitivity, which results in acute burning and painful sensations (without blistering) upon exposure to sunlight [1]. Around 20–30% of individuals with EPP are also at risk for hepatic manifestations, including cholelithiasis (about 20%), liver disease (1–4%), and (rarely) liver failure requiring transplant [1]. Biochemical analysis of affected individuals reveals high levels of free protoporphyrin in plasma or erythrocytes (>2000 μg/dl, with a normal range of 20–80 μg/dl), as well as detection of a characteristic 634 nm fluorescent peak in plasma. A comparison of free protoporphyrin and zinc protoporphyrin levels can help distinguish EPP from XLP. The zinc protoporphyrin level is low in EPP cases, but is typically around 40% in XLP cases [1].

## 2 Results/discussion

**Fig. 1:**
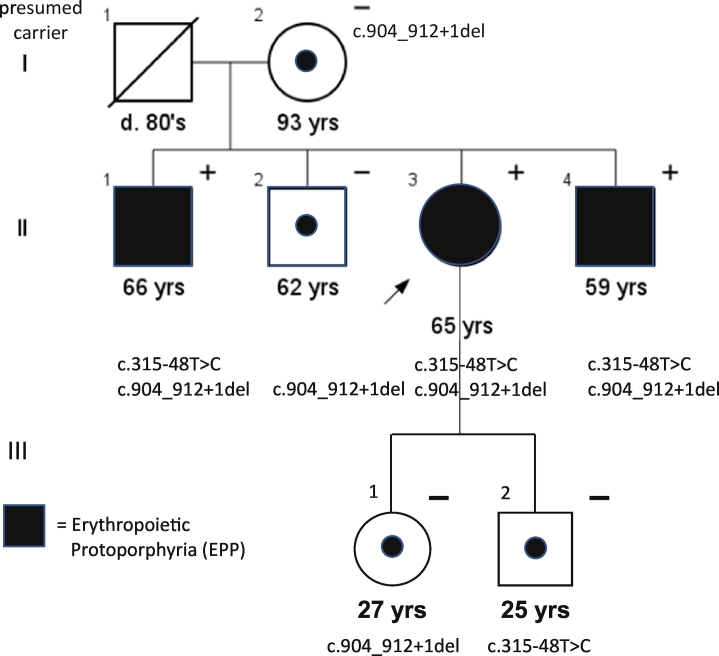
Pedigree and genetic status of study family affected by EPP.

We report a family of northern European ancestry with three out of four siblings affected with EPP (Fig. 1).

All were diagnosed at a young age based on photosensitivity and family history. All are currently in middle age and have known about their diagnosis for many years, but were interested in understanding the genetic basis of their disease as well as the inheritance pattern. The three affected individuals experience the classic cutaneous features of EPP: immediate painful photosensitivity with burning sensation, edema, and petechiae but no blistering. The duration of symptoms is typically a few days, although the proband reported once missing a week of work in her youth after unintentional sun exposure during the winter. None of the patients reported liver manifestations. Recent liver function studies revealed results within the normal range for the proband and both affected siblings (Table 1). One sibling (II:1) has mild liver fibrosis as noted via elastography.

**Table 1:** Biochemical results for EPP study family.

Subject	Protoporphyrin μg/dl (range 20–80)	% Zinc protoporphyrin, % Free protoporphyrin	Liver function studies⁎
II:1	2644	6, 94	Normal liver panel; mild liver fibrosis
II:2	100	67, 33	No data
II:3	2045	4, 96	Normal liver panel
II:4	2845	5, 95	Normal metabolic panel (alk phos, bilirubin, albumin, total protein)
III:1	92	74, 26	No data

⁎ Alkaline phosphatase, albumin, ALT, AST, bilirubin (total and direct), total protein.

Biochemical studies measuring protoporphyrin levels were consistent with expected levels for affected versus unaffected family members (Table 1). The three affected siblings showed very elevated levels of protoporphyrin (>2000 μg/dl), while heterozygous carriers of one variant showed only slightly elevated levels (ranging from 90 to 100 μg/dl). The proband and one other affected sibling tested positive for the characteristic 634 nm peak in plasma (data not shown). Finally, the ratio of zinc protoporphyrin to free protoporphyrin in affected family members is low, consistent with EPP (Table 1).

Seven family members have undergone genetic testing (Fig. 1), including the proband (II:3), her three brothers (II:1, 2, 4), their mother (I:2), and the two children of the proband (III:1, 2). All three siblings with EPP were heterozygous for the common c.315-48T>C hypomorphic variant and a previously undescribed in-frame deletion designated c.904_912+1del (p.Gln302_Lys304del) (Fig. 1). Segregation of each variant in the proband's children (III:1, 2) shows that the two variants are in *trans*. The c.904_912+1del variant has not been reported in the literature, in public variant databases, nor in the Porphyrias Consortium mutation database curated by the laboratory of Dr. Robert Dresnick at the Icahn School of Medicine at Mount Sinai (pers. com.). Since the c.904_912+1del variant is predicted to result in loss of three codons and spans a splice donor site, additional studies were conducted to further characterize the impact of the c.904_912+1del variant on gene function. This in-frame deletion may either result in the loss of three amino acids, or due to its proximity to a splice junction it may impair mRNA splicing and ultimately protein translation. As the c.904_912+1del variant spans a splice junction, we used five different splicing prediction programs to determine if aberrant splicing is a potential mechanism of pathogenicity [2].

**Fig. 2:**
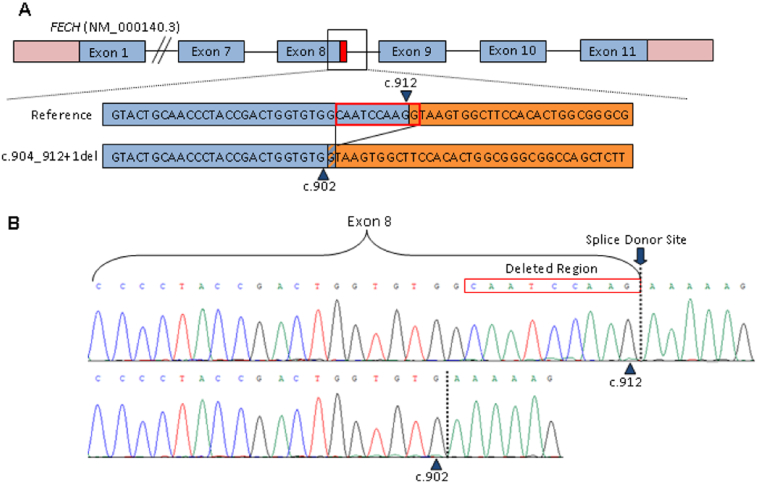
Functional studies of the c.904_912+1del variant using a minigene splicing assay. **A**) Splice site prediction algorithms support the canonical splice donor site at c.912 for the wild type allele. The *FECH* c.904_912+1 deletion is predicted to create a cryptic splice donor site at c.902, resulting in a frameshift and premature FECH protein termination (p.Gln302Leufs*31). ▲ = predicted splice donor sites. **B**) Sanger sequencing of *FECH* cDNA minigene studies support splice site predictions, with a splice donor site observed at c.912 for the wild type allele, and a splice donor site at c.902 for the *FECH* c.904_912 + 1 deletion allele. Dashed vertical lines indicate sites of splicing.

Functional studies revealed that the c.904_912+1del variant results in the deletion of the canonical splice donor site at the exon 8/intron 8 boundary of the *FECH* gene, therefore causing aberrant mRNA splicing leading to a frameshift and premature protein truncation. This is in contrast to the possibility of an in-frame deletion of three codons (deletion of three amino acids) with normal splicing mechanisms intact. Splicing prediction programs (SpliceSite Finder-like, MaxEntScan, and NNSPLICE-Alamut v.2.11) indicated that this deletion activates a cryptic splice donor site at c.902 in exon 8. This change could result in a frameshift and premature protein truncation (p.Gln302Leufs*31) if splicing proceeded with the canonical splice acceptor site at the intron 8/exon 9 boundary (Fig. 2A).

A previously described minigene splicing assay was used to further assess potential splicing effects of the c.904_912+1del variant [3,4]. Briefly, wild-type *FECH* exon 8 gene sequence with flanking intronic sequence or the c.904_912+1del variant were amplified from patient DNA and cloned into the pRHCglo vector using *SalI* and *XbaI* restriction sites. Plasmid constructs were transfected into 3T3 murine embryonic fibroblast cells. RT-PCR was performed on RNA extracted from the cells. Two replicates of cDNA from the wildtype and mutant transfected cells were sequenced and aligned for comparison. *FECH* cDNA sequencing confirmed a functional canonical splice donor site at c.912, which is consistent with typical *FECH* mRNA processing at the exon 8/intron 8 boundary. *FECH* cDNA sequencing from cells transfected with the mutant plasmid construct revealed splicing had occurred at c.902, the location of the cryptic splice donor site predicted by SpliceSite Finder-like, MaxEntScan, and NNSPLICE algorithms (Fig. 2B). Taken together, these data support that the c.904_912+1del variant results in the activation of a cryptic splice donor site in exon 8 of the *FECH* gene, which in turn causes aberrant splicing and truncation of the FECH protein.
